# Supplementary material for: Association between oral microbiome and seven types of cancers in East Asian population: a two-sample Mendelian randomization analysis
Source: Front Mol Biosci. 2023 Nov 21;10:1327893. doi: 10.3389/fmolb.2023.1327893 (PMC10702768; doi:10.3389/fmolb.2023.1327893)
Supplement: Supplementary file 1 [file DataSheet1.ZIP › Supplementary Materials/MR plots for tongue/tongue═╝/Gastric cancer/pheno.2785_to_gastric cancer_forest.pdf]

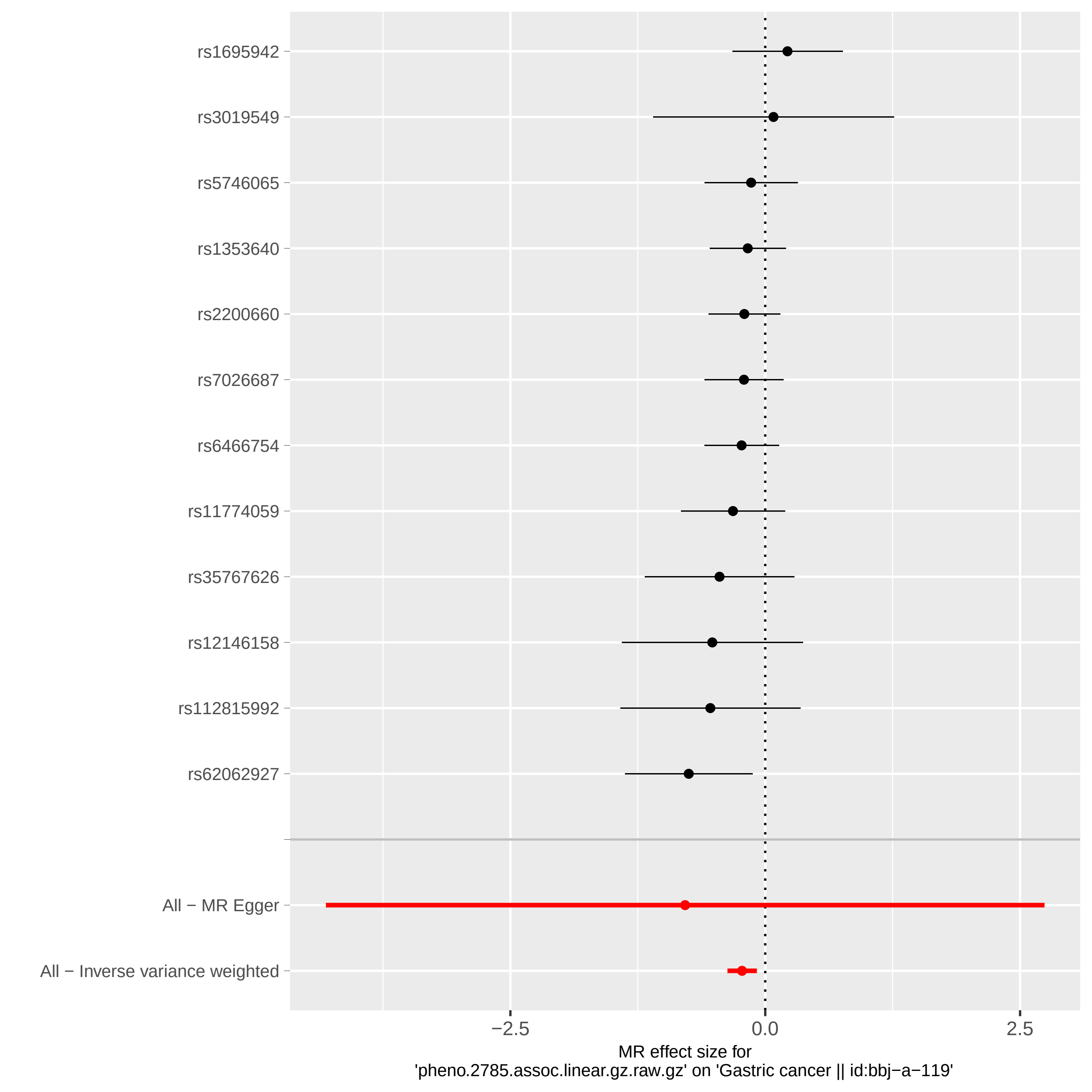

rs1695942
rs3019549
rs5746065
rs1353640
rs2200660
rs7026687
rs6466754
rs11774059
rs35767626
rs12146158
rs112815992
rs62062927
All − MR Egger
All − Inverse variance weighted
−2.5
0.0
2.5
MR effect size for
'pheno.2785.assoc.linear.gz.raw.gz' on 'Gastric cancer || id:bbj−a−119'
